# The Effect of the Argon Carrier Gas in the Multiphoton Dissociation-Ionization of Tetracene

**DOI:** 10.3390/ijms9102003

**Published:** 2008-10-28

**Authors:** Juan Carlos Poveda, Alejandro San Román, Alfonso Guerrero, Ignacio Álvarez, Carmen Cisneros

**Affiliations:** Laboratorio de Colisiones Atómicas Moleculares, Instituto de Ciencias Físicas, Universidad Nacional Autónoma de México, Av. Universidad Colonia Chamilpa, Cuernavaca, Morelos C.P. 62210, México. E-Mails: asanroman@fis.unam.mx (A. R.); alfonsog@fis.unam.mx (A. G.); ialvarez@fis.unam.mx (I. Á.); carmen@fis.unam.mx (C. C.)

**Keywords:** Polycyclic Aromatic Hydrocarbons, Tetracene, Multiphoton dissociation, ToF-MS

## Abstract

The multiphoton dissociation-ionization of tetracene at 355 nm using 6.5 nanosecond laser pulses, with and without argon as a carrier gas (CG), has been studied and compared. Ion fragments were analyzed in a time-of-flight mass spectrometer and separated according to their mass-to-charge ratio (*m/z*). The results show that the dynamic of photodissociation at ∼10^10^ W cm^−2^ intensities is strongly influenced by the CG. The suppression of fragmentation channels primarily those relating to the formation of the CH_m_^+^ (m = 2, 4), C_2_H_4_^+^ and C_5_H_4_^+2^ ions. CH_5_^+^ and CH_6_^+^ were observed which have not been reported before in photodissociation tetracene experiments.

## 1. Introduction

Polycyclic aromatic hydrocarbons (PAH’s), such as tetracene (C_18_H_12_) are generated primarily by combustion processes, and constitute the largest single class of chemical carcinogens known today [1, 2]. In addition to their terrestrial importance, extensive observational and experimental work over the past decades has indicated that PAH’s are the dominant class of molecular species in some regions of the interstellar medium [3–9]. Neutral and ionized PAH’s have been proposed as the molecules responsible of an ubiquitous family of infrared emissions (UIR) observed from various interstellar objects [3, 4] and they have also been considered as unstable systems under intense radiation fluxes, thus the contribution of dehydrogenated or fragmented PAH’s to the UIR emissions may be very important, especially in stellar regions such as at the inside interface with HII or H^+^ regions and in the central regions of planetary nebulae [7].

Molecular ionization and dissociation induced by intense laser radiation has been the subject of increasing interest in recent years [10]. The mechanisms which are responsible for molecular ionization and dissociation are strongly related to the wavelength and intensity of laser radiation fields. It has been shown that with laser intensities higher than 10^8^ W cm^−2^ the irradiation with nanosecond, (ns) pulse widths often leads to molecular fragmentation through simultaneous absorption of several photons [10–12]. However, as intensity increases up to values of 10^11^ W cm^−2^ and above the use of picosecond (ps) and femtosecond (fs) pulses leads to intact molecular ion production, which is absent in the ns laser-induced mass spectra [10–13] at the present wavelength. For intensities higher than 4×10^13^ W cm ^2^ the ionization process can even take place through a field ionization mechanism and/or multiphoton processes [10, 14, 15]. In the ns laser pulse regime the order of the photoion yield as a function of laser intensity is a common predictor of ionization mechanism. In the case of multiphoton ionization a plot of the logarithm of the ion yield versus the logarithm of the laser power typically yields a line whose slope is equivalent to the number of photons necessary to reach either the ion state or an intermediate, long-lived eigenstate of the molecule according to relation:

(1) $$Y=\sigma_{n}I^{n}$$

where *Y* is the ion yield, σ*_n_* is the *n*^th^ order cross section in units commensurate with the units used for intensity, and *n* is the order of the process [15].

In this paper we report the multiphoton dissociation and ionization of tetracene induced by intense laser radiation over a wide energy/pulse interval (∼10^10^ W cm^−2^). We have been interested mainly in the effect of the carrier gas (CG) on the photodissociation pattern, the relative and total photoion yield and the energy absorbed to produce the different ion fragments. The experiments were conducted using the third harmonic of a nanosecond Nd:YAG laser system coupled to a time-of-flight mass spectrometer.

## 2. Experimental

The experimental setup used has been described in detail elsewhere [16]. It consists of a time-of-flight (ToF) mass spectrometer, a supersonic molecular beam, and a Nd:YAG laser (Quanta Ray, Spectra Physics). The ToF mass spectrometer is used in a conventional linear arrangement with ion optics based on a Wiley-McLaren design [17]. It consists of one vacuum chamber coupled to a linear ToF-MS system. It is pumped by two turbomolecular pumps (Leybold, Turbovac 450) with a pumping rate of 450 L/s, both backed up by an oil-free scroll pump (Alcatel, Drytel 31). The pressures in the chambers were maintained at about 2.7×10^−9^ kPa of ground pressure, and 2.7×10^−7^ kPa during the experiments.

The tetracene samples (99%, Aldrich Chemical Co.) were admitted from the inlet system (thermal chamber) toward the mass spectrometer interaction region (base pressure of 2.7×10^−9^ kPa) through an electromagnetic valve (nozzle) and a skimmer. Neutral tetracene vapor (produced by heating high-purity tetracene at ∼330 °C) was used under two experimental conditions: with argon gas as CG and without it, at typical backing pressure of 34.5 kPa. The samples passed and expanded through a 0.8 mm diameter nozzle in pulsed mode with 200 μs of aperture, coupled synchronously with the laser pulse using a controller (Iota One, General Valve). Thus the expansion conditions were reached to form a supersonic beam which was directed perpendicularly to the pulsed laser beam.

The ion fragments were extracted by an electrode-mesh polarized to +5.0 kV and subsequently accelerated by a repulsive voltage of +3.5 kV toward a grounded grid electrode at the entrance of the 1 meter long field free flight tube. The positive ions were detected by a channel electron multiplier (Sjuts KBL 10RS), and signal was amplified by a fast preamplifier (Ortec VT120) and subsequently digitized using a multichannel scaler (Turbo-MCS, EG&G ORTEC) to reconstruct the ToF mass spectra in a ToF windows of 20 μs, 1,000 channels, 20 ns per channel; typically 5,000 of laser shots for each spectrum were added.

The laser radiation was obtained from the third harmonic of the Nd:YAG laser corresponding to pulses of 355 nm, with a 6.5 ns duration, a 10 Hz repetition frequency and energies at about 20–100 mJ/pulse. The laser beam was focused into the interaction region, 12 cm from the nozzle of the ToF mass spectrometer using a convex lens with a 150 mm focal length producing an intensity about 1.1×10^10^-6.40×10^10^ W cm^−2^. The intensity values in this work have been estimated by considering the energy/pulse, pulse width and the measured focal area, ∼240 μm^2^.

## 3. Results and Discussion

### 3.1. Photodissociation/photoionization of Tetracene without CG

**Figure 1.**
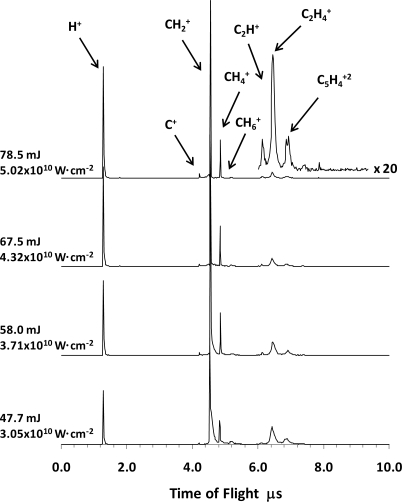
Photodissociation ToF spectra at 355 nm, without carrier gas.

The ToF mass spectra of tetracene without carrier gas that were obtained using 355 nm laser radiation in the nanosecond regime (6.5 ns pulse width) and intensities of ∼10^10^ W cm^−2^ consist of ion fragments whose *m/z* is relatively low, 1 ≤ *m/z* ≤ 32. The photofragmentation pathways lead to the formation of the H^+^, C^+^, CH_m_^+^(m = 2, 4, 5, 6), C_2_H_m_^+^ (m = 1,4) single charged ions and C_5_H_4_^+2^ doubly charged ion. Some of the ToF spectra as a function of the laser energy are shown in Figure 1 and the main products of the molecular fragmentation are given in Table 1. At low laser radiation intensities the relative abundances of the H^+^, CH_2_^+^, CH_6_^+^, C_2_H_4_^+^ and C_5_H_4_^+2^ ions represent about 90% of the total yield, while at laser energies higher than 45 mJ/pulse (2.88×10^10^ W cm^−2^) the mass spectra are dominated mainly by the H^+^, CH_m_^+^ (m = 2, 4) and C_2_H_4_^+^ ions. whose joint contribution to the total yield is about 90%. The most outstanding characteristics of the ToF mass spectra are an extensive molecular fragmentation; the production of doubly charged ions and absences of the parent molecular, C_18_H_12_^+^, and acetylene, C_2_H_2_^+^, ions. The formation of acetylene has been previously reported in ionization and dissociation studies of tetracene carried out using ionization by electron impact [7, 18, 19] and ionization using VUV single photons [20], nevertheless its formation is not observed in typical nanosecond and femtosecond multiphoton ionization experiments [21–23].

The extensive fragmentation of tetracene and the absence of the parent ion M^+^ that were observed in the mass spectra at 355 nm agree with experimental studies carried out previously [10, 12, 24] on a wide variety of polyatomic molecules using UV, visible and infrared laser radiation pulses on the ns scale and intensities higher than 10^8^ W cm^−2^. Under such conditions the molecules are fragmented through a process of dissociation followed by ionization, *D-I*, which is characterized by extensive multiphoton fragmentation, so that small fragments dominate the mass spectra at the expense of parent ions. In contrast, Robson *et al*. [21–23] have studied the ionization and fragmentation of tetracene using femtosecond and nanosecond laser pulses over a wide range of intensities. The spectra obtained with 70–80 fs pulses and intensities of 10^14–15^ W cm^−2^ exhibit strong parent ion peaks, doubly and triply charged parent ions and doubly charged hydrocarbon groups, C_m_H_n_^+2^. In addition, the C_m_^+^ group and a strong H^+^ peak are present in the mass spectra, in apparent agreement with our results; nevertheless, the ionization and fragmentation are attributed to a mechanism of field ionization and/or multiphoton process. For the ns mass spectra, the parent ion dominates the spectra when UV photons are used at lower intensities than 10^8^ W cm^−2^, but as the intensity increases the formation of low mass fragments increases, particularly the C_m_^+^, C_3_H_m_^+^ and C_6_H_m_^+^ groups were observed and no multiply charged ion was produced, which differs considerably with our results, however Robson *et al*. [22] have used 266 nm, 16 ns laser radiation. There is also a very recent study on multiphoton ionization of PAH′s using 266 nm laser radiation [25]; after they are desorbed by laser interaction in this experiment, depending on the laser intensity, parent ions, aromatic fragments C_n_H_m_^+^ with (n= 10–2) and atomic carbon and hydrogen were observed and a detail discussion on the dissociation mechanisms is presented.

A relevant aspect in the ToF mass spectra reported here is the production of the ions appearing at *m/z*=17 and 18, ToF of 5.01, and 5.16 μs, respectively, which has not been observed in fragmentation processes of tetracene previously published by others authors [7, 18–23]. These ions might be generated from the methane ion, ^13^CH_4_^+^ or deuterated methane, CH_3_D^+^, ^13^CH_3_D^+^, and CH_2_D_2_^+^, nevertheless their yields represent a large percentage of the yield, approximately of 6–50%, in disagreement with contributions only from ^13^C or deuterium, whose isotopic abundances are 1.1 and 0.01% respectively. Therefore, we have considered that the ions of *m/z* = 17 and 18 in the spectra correspond principally to protonated methane ions, CH_5_^+^ and CH_6_^+^. Asvany *et al.* [26] proposed that the formation of CH_5_^+^ is through CH_6_^+^, an intermediary complex, coming from the H_2_ addition to CH_5_^+^. The short life of CH_6_^+^, is explain as the result of its fast dissociation leading to CH_5_^+^ + H. In our experiments CH_6_^+^ was detected and its lifetime was derived from ToF, been longer than 5.21 μs, even though its structure is still under discussion. In order to find the possible structures we carried out DFT and MP_n_ calculations and found three different stable geometries. *Ab-inito* calculations had been reported [27, 28] about the stability of the multiple charged ions: CH_6_^+2^, CH_6_^+3^, and CH_6_^+4^.

We propose that these ions can be formed in a plasma by a growing mechanism from the simple ions, CH^+^, H^+^, H and H_2_, and can be seen as follows:

(2) $$\begin{array}{l} {\text{CH}}^{+}+{\text{H}}_{2}\;\to {\text{CH}}_{3}^{+}\\ {\text{CH}}_{3}^{+}+{\text{H}}_{2}\;\to {\text{CH}}_{5}^{+} \end{array}$$

and

(3) $$\begin{array}{l} {\text{CH}}_{2}^{+}+{\text{H}}_{2}\;\to {\text{CH}}_{4}^{+}\\ {\text{CH}}_{4}^{+}+{\text{H}}_{2}\;\to {\text{CH}}_{6}^{+} \end{array}$$

The observed onset energy for the photoionization and photodissociation of tetracene is of ∼ 22 mJ (1.41×10^10^ W cm^−2^). From this energy the yield of all detected fragments undergo an increase as the laser intensity increases according to equation (1). Nevertheless, when the laser intensity is increased to values greater than 2.88×10^10^ Wcm^−2^ (45 mJ/pulse), two important aspects can be noticed in the ToF mass spectra: (*i*) The relative yield of the ions, *m/z* ≥ 18, that were observed diminish and the relative yield of the CH_m_^+^ ions increases; (*ii*) once the onset of tetracene fragmentation is attained the distribution pattern remains unaltered as the intensity increases, *i.e*. is not observed the formation of different ion fragments. Then it is probable that the larger *m/z* ions, *m/z* ≥ 17, undergo further fragmentation processes which yield to the CH_2_^+^ and CH_4_^+^ ions in high relative abundance. The joint contribution of the larger *m/z* ions, *m/z* ≥ 17, to the total ion yield decreases by ∼20% to energies higher than 45 mJ/pulse, meanwhile the relative yield of the CH_2_^+^and CH_4_^+^ ions increases by 15% and 5% respectively, and that of H^+^ and C^+^ ions is held almost constant, which suggest that main channels involved in the production of the CH_m_^+^ (m = 2, 4) ions might be by the dissociation of the C_5_H_4_^+2^, C_2_H_4_^+^, C_2_H^+^ and CH_5_^+^ ions. In order to get a better understanding of results we have estimated the order of the ionization process (the number of absorbed photons *n* to produce the ion fragments).

In accord with Equation 1, a plot of the logarithm of the photoion yield versus the logarithm of the laser energy was made for each ion and the order of the process, *n*, was calculated. The order of the processes multiplied by the energy of the 355 nm photons, are included, along with the results obtained with carrier gas, in columns 1 and 2 of Table 1. The C_2_H_4_^+^, CH_5_^+^, CH_4_^+^ and CH_2_^+^ ions result from absorption processes of 3.7, 2.5, 4.4 and 5.7 photons, respectively, these values support the interpretation of our results, mainly the fragmentation mechanisms of C_2_H_4_^+^ and CH_5_^+^ ions to produce the CH_2_^+^ and CH_4_^+^ ions by the additional absorption of more photons, i.e.:

(4) $$\begin{array}{l} {{\text{C}}_{2}\text{H}}_{4}^{+}+h\nu \;\to {\text{C}\text{H}}_{4}^{+}+\text{C}\\ {{\text{C}}_{2}\text{H}}_{4}^{+}+2h\nu \;\to {\text{CH}}_{2}^{+}+{\text{CH}}_{2}^{+}\\ {\text{CH}}_{5}^{+}+2h\nu \;\to {\text{CH}}_{4}^{+}+\text{H}\\ {\text{CH}}_{5}^{+}+3h\nu \;\to {\text{CH}}_{2}^{+}+\text{3H} \end{array}$$

It is also possible that many other dissociation channels are involved in their production. The non-integer orders, *n*, that we have measured (Table 1), might be taken as tentative evidence of the coexistence of multiple fragmentation channels. In addition, according to various authors [29, 30], different photoions that come from a common precursor will have the same laser intensity dependence, *I^n^*, so that the yield of H^+^ and C^+^ ions, which result from a absorption of 2.2 photons, ∼7.7 eV, is likely through the same precursor ion, for example the fragmentation of the C_2_H^+^ and CH_5_^+^ ions, from high excited states, could enhance the H^+^ and C^+^ ion yield and reduce their own relative abundances through the following processes:

(5) $$\begin{array}{l} {\text{(C}}_{2}{\text{H}}^{+}\text{)}*\to {\text{H}}^{+}+{\text{C}}_{2}\\ {({\text{C}}_{2}{\text{H}}^{+}\text{)}}^{*}\to {\text{C}}^{+}+\text{CH}\\ {{(\text{CH}}_{5}^{+}\text{)}}^{*}\to {\text{H}}^{+}+{\text{CH}}_{4} \end{array}$$

### 3.2. Effect of the argon CG

**Figure 2.**
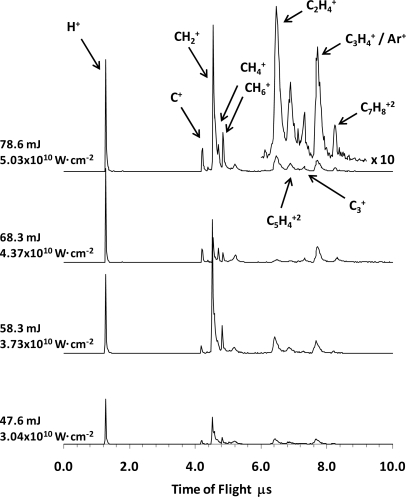
PD - ToF spectra at 355 nm, with carrier gas.

Some of the ToF mass spectra of tetracene obtained with CG under identical laser radiation conditions (355 nm and 6.5 ns laser radiation pulses) are plotted as a function of the energy (80–94 mJ/pulse) in Figure 2.

The main produced ions are H^+^ and C_3_H_4_^+^ or Ar^+^; they represent about 60% of the total yield in the whole interval of energies used (74–100 mJ/pulse); the CH_6_^+^, and C_5_H_4_^+2^ and C_7_H_8_^+2^ doubly charged ions account for about 15%; and the relative abundances of the C_m_^+^ (m = 1, 3) and CH_m_^+^ (m = 1–5) groups are of 10% and 12% respectively. The absorbed number of photons are given in Table 1, colum 4.

The ToF spectra differ from those presented in the previous section, where was observed that tetracene photodissociation channels lead mainly to the production of the CH_2_^+^, CH_4_^+^, C_2_H_4_^+^, C_5_H_4_^+2^ and H^+^ ions, and that the further fragmentation of the larger *m/z* detected ions enhances the production of the smaller mass ions. The joint yield of the CH_2_^+^, CH_4_^+^, C_2_H_4_^+^ and C_5_H_4_^+2^ ions represents about 11% and 76–85% of the total yield in the spectra with and without CG, respectively. In addition, the relative yield of the ions with 12 ≤ *m/z* ≤ 32 is maintained almost constant, and the yield of the greater mass ions, 36 ≤ *m/z* ≤ 46, is enhanced when the laser intensity is increased from 74 to 100 mJ/p, particularly the relative abundances of the C_3_^+^, C_3_H_4_^+^ and C_7_H_8_^+2^ ions increase from 50% to almost 65%.

**Figure 3.**
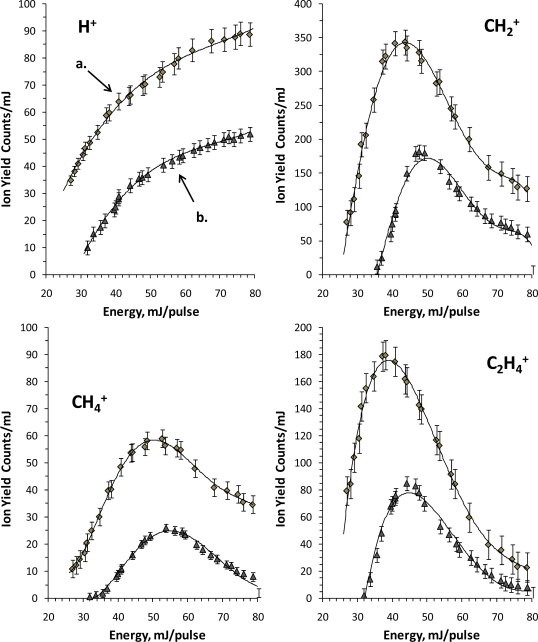
Comparative carrier gas effect in *PD* of tetracene at 355 nm: a) without carrier gas; b) with argon as carrier gas.

**Figure 4.**
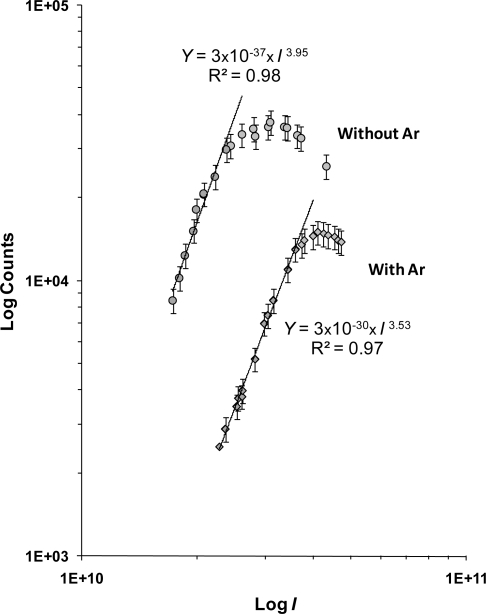
Log-Log plots of the total ion currents of tetracene with or without carrier gas.

Clearly the kind of fragmentation experienced by the tetracene as well as the intensity of the photoion yield are strongly influenced by the CG (Figures 1 and 2), which induces the formation of a wide variety of fragments, some of which are of higher *m/z*, doubly charged, and are not observed in the mass spectra without CG, *e.g*. CH^+^, CH_3_^+^, C_3_^+^, C_3_H_4_^+^ and C_7_H_8_^+2^. The normalized ion yield to the energy for some of the ion fragments as a function the laser energy are plotted in Figure 3 where a shift of the maximum towards higher energies can be observed for the formation of a particular ion and a decrease in the signal intensity as a result of the CG. The observed onset energy for the fragmentation is higher than the observed onset in the fragmentation without CG. In addition, the total yield without CG apparently tends towards a saturation level at energies higher than 45 mJ, 2.88×10^10^ W cm^−2^, which has not been observed with CG within this energy range, see Figure 4. According to our results it is likely that the observed features at the spectra with CG are due to: (*i*) possible recombination processes of smaller mass neutral photoions which could reduce the intensity of the ion yield and promote the diversity not only of a wide variety of fragments, but also the production of ions larger *m/z* (*e.g*. 2CH_2_ + C → C_3_H_4_ → C_3_H_4_^+^; C + H → CH → CH^+^); (*ii*) tetracene could access to new channels of molecular dissociation which enhance largely the production of the C_3_^+^, C_3_H_4_^+^ and C_7_H_8_^+2^ photoions in the used intensity region; and (*iii*) collisions between photoions of tetracene and CG atoms might disable or prevent the dissociation of the heavy mass fragments (as the fragmentation that was observed in the spectra of tetracene without CG) mainly the dissociation and ionization channels leading to the production of the CH_2_^+^ and CH_4_^+^ ions, as well as photodissociation channels involved in the production of the C_5_ H_4_^+2^ and C_2_ H_4_^+^ ions. However, it is important to mention on the need to develop a larger number of studies to understand in a more decisive manner the effects caused by the argon CG.

Finally, the measured orders of the H^+^ and C^+^ ions are almost exactly those calculated from the multiphoton dissociation-ionization of tetracene without CG (Table 1); in both photodissociation processes about 2.0 to 2.2 photons, ∼7.3 eV, are absorbed for their production. This suggests that argon CG does not affect the photo-absorption processes involved in the formation of the ions before mentioned. The small difference between the measured orders from the total ion yield of tetracene with and without CG, 3.53 ± 0.07 and 3.95 ± 0.07 photons respectively (see Figure 4) is consistent with the fact that main contributions to the total yield are due to: the absorption processes of 2.0 (H^+^) and 4.2 (C_3_ H_4_^+^) photons in the fragmentation with CG; and the absorption of 2.2 (H^+^), 5.7 (CH_2_^+^), 4.4 (CH_4_^+^), 3.7 (C_2_ H_4_^+^) and 2.9 (C_5_ H_4_^+2^) photons in the corresponding without CG. The number of absorbed photons and the energy to produce some of the remaining ion fragments of tetracene with CG can be found Table 1. As was reported by Troxler [31, 32], the interaction of argon with PAH′s, naphthalene, results in a progressive red shift in the energy of electronic levels of molecule. The very weak tetracene-argon interaction can be neglected as photons are absorbed. In our experiments the major effect was observed in the order of processes, n, of protonated carbon ions, CHn^+^, been notably lower than that the observed without carrier gas.

## 4. Conclusions

The multiphoton fragmentation of tetracene at 355 nm has been carried out using a time-of-flight mass spectrometer coupled to a nanosecond Nd:YAG laser system. The ToF mass spectra were characterized by the absence of the parent molecular ion and an extensive fragmentation, with lower mass fragments, over the whole energy/pulse interval used, ∼10^10^ W cm^−2^. The main fragmentation pathways lead to the formation of the H^+^, C^+^, CH_m_^+^ (m = 2, 4, 5, 6), C_2_H_m_^+^ (m = 1, 4) single ions and C_5_H_4_^+2^ doubly charged ions. The photoion yield has been measured as a function of the laser intensity. The intensity dependences, *n*, are different for the ion fragments indicating that tetracene dissociation followed by ionization, D-I, is the most likely mechanism. This process is due predominantly to the 1.9–5.7 photon absorption. The results presented reveal that the D-I processes is a “ladder-switching” type mechanism [33] as it has been observed before in other PHA studies [25].

The photodissociation dynamic in the laser intensity range used is strongly influenced by the CG; this one has a significant impact on the type of ion fragments formed as well as in their relative abundances. Particularly, has been observed a decrease of the relative and total ion yield and a shift of the maxim toward higher laser energies for the formation of a particular ion, the suppression of some dissociation channels related to the formation of the CH_m_^+^ (m = 2,4), C_2_H_4_^+^ and C_5_H_4_^+2^ ions, the production of the CH_m_^+^ (m=1,3) ions and heavier mass ions: C_3_^+^, C_3_H_4_^+^ single ions and C_7_H_8_^+2^ doubly charged ion, which might be result of possible recombination processes of small mass neutral and charged photoions or as a consequence of opening new molecular dissociation channels from tetracene. Finally, the measure of the photoion yield as a function of the laser energy indicated that the dissociation-ionization process is due to the absorption of 2.0 up to 5.1 photons, which does not differ significantly with the fragmentation process of tetracene without CG. The present results shed some light on the behavior of the PAH’s interacting with laser radiation, which in turn is very important for combustion and environmental studies.

## Figures and Tables

**Table 1.** Calculated relative ion yield of *PD* of Tetracene.

Ion	Without C.G.		With C.G.	
	n	n·hν, eV	n	n·hv, eV
H^+^	2.23±0.22	7.78	2.02 ± 0.20	7.05
C^+^	2.20±0.22	7.68	2.24 ± 0.22	7.82
CH_2_^+^	5.74±0.57	20.03	3.60 ± 0.36	12.56
CH_3_^+^	---	---	3.50 ± 0.35	12.22
CH_4_^+^	4.40±0.44	15.36	3.36 ± 0.34	11.73
CH_5_^+^	2.55±0..26	8.90	4.10 ± 0.41	14.31
CH_6_^+^	---	---	3.02 ± 0.30	10.54

C_2_H^+^	1.92±0.19	6.70	---	---
C_2_H_4_^+^	3.72±0.37	12.98	1.63 ± 0.16	5.69

C_3_^+^	---	---	3.11 ± 0.31	10.85
C_3_H_4_^+^	---	---	4.20 ± 0.42	14.66

C_5_H_4_^+2^	---	---	3.72 ± 0.37	12.98

C_7_H_8_^+2^	---	---	5.10 ± 0.51	17.80

Experimental error = 10%.
